# Drug–Drug Interactions Involving Dexamethasone in Clinical Practice: Myth or Reality?

**DOI:** 10.3390/jcm12227120

**Published:** 2023-11-15

**Authors:** Venceslas Bourdin, William Bigot, Anthony Vanjak, Ruxandra Burlacu, Amanda Lopes, Karine Champion, Audrey Depond, Blanca Amador-Borrero, Damien Sene, Chloe Comarmond, Stéphane Mouly

**Affiliations:** 1 Internal Medicine Department, Département Médico-Universitaire INVICTUS, Lariboisière Hospital, Assistance Publique-Hôpitaux de Paris (APHP).Nord—Université Paris-Cité, 75010 Paris, France; venceslas.bourdin@aphp.fr (V.B.); william.bigot@aphp.fr (W.B.); anthony.vanjak@aphp.fr (A.V.); ruxandra.burlacu@aphp.fr (R.B.); amanda.lopes@aphp.fr (A.L.); karine.champion@aphp.fr (K.C.); audrey.depond@aphp.fr (A.D.); blanca.amador-borrero@aphp.fr (B.A.-B.); damien.sene@aphp.fr (D.S.); chloe.comarmondortoli@aphp.fr (C.C.); 2 INSERM U976, Hôpital Saint-Louis, 75010 Paris, France; 3 INSERM UMR-S1144, Hôpital Fernand Widal, 75010 Paris, France

**Keywords:** dexamethasone, drug–drug interaction, cytochrome P450 (CYP) 3A4, P-glycoprotein(P-gp), pharmacokinetics, side effect

## Abstract

Concomitant administration of multiple drugs frequently causes severe pharmacokinetic or pharmacodynamic drug–drug interactions (DDIs) resulting in the possibility of enhanced toxicity and/or treatment failure. The activity of cytochrome P450 (CYP) 3A4 and P-glycoprotein (P-gp), a drug efflux pump sharing localization and substrate affinities with CYP3A4, is a critical determinant of drug clearance, interindividual variability in drug disposition and clinical efficacy, and appears to be involved in the mechanism of numerous clinically relevant DDIs, including those involving dexamethasone. The recent increase in the use of high doses of dexamethasone during the COVID-19 pandemic have emphasized the need for better knowledge of the clinical significance of drug–drug interactions involving dexamethasone in the clinical setting. We therefore aimed to review the already published evidence for various DDIs involving dexamethasone in vitro in cell culture systems and in vivo in animal models and humans.

## 1. Introduction

Concomitant administration of multiple drugs frequently causes severe pharmacokinetic or pharmacodynamic drug–drug interactions (DDIs) resulting in the possibility of enhanced toxicity and/or therapy failure. A DDI occurs when the effects of one drug are changed by the presence of another drug, food or some environmental factor. Pharmacokinetic DDI may be divided into two categories, i.e., induction and inhibition of enzymes and transporters involved in drug metabolism and transport. The induction and inhibition of such enzymes and/or transporters may result in decreases or increases in the blood concentration of a drug, thus modifying the drug’s effects. Important advances in the knowledge of human drug-metabolizing enzymes have fueled the integration of in vitro drug metabolism and clinical DDI studies for use in drug development programs and in the clinical setting.

The activity of cytochrome P450 (CYP) 3A4 and P-glycoprotein (P-gp), a drug efflux pump sharing localization and substrate affinities with CYP3A4 is a critical determinant of drug clearance, interindividual variability in drug disposition and clinical efficacy, and appears to be involved in the mechanism of numerous clinically relevant DDIs. Usually, the risk of significant DDI increases when a patient requires numerous medications, higher doses of medication and a longer duration of therapy, as observed in many patients who may be candidates to be treated with dexamethasone, classically described as a CYP3A4 and P-gp substrate and modulator [1].

The current literature review is based on published citations on PUBMED and EMBASE, between 1967 and May 2023, using “dexamethasone”, “Drug”, “Interaction”, “P-glycoprotein” and/or “transporters” as keywords. We aimed to review the already published evidence for various DDIs involving orally or intravenously administered dexamethasone, identified in vitro in cell culture systems and in vivo in animal models and humans, mostly healthy volunteers. The current revised non-clinical and clinical overview of DDIs involving dexamethasone focused on their respective mechanisms as well as the respective role of liver and small intestinal CYP3A4 and transporters in the occurrence of such DDIs, together with the ability of in vitro tools to accurately predict the in vivo consequences of the identified or putative DDIs with dexamethasone.

## 2. Dexamethasone, an Increasingly Used Molecule

Apart from the use of -dose dexamethasone in endocrinologic diagnosis tests [2], this drug has mainly been used at high dosages in hematologic malignancy multi-drug protocols, especially in multiple myeloma [3], but also in lymphoma, most often as a second-line treatment; except in the management of mantle cell lymphoma, where dexamethasone is used as a first-line therapy [4]. Over the past 3 years, its utilization has, however, somewhat changed. The COVID-19 pandemic has dramatically increased the use of high-dose dexamethasone outside of the scope of hematologic malignancies, with proven efficacy in patients with severe COVID-19 infection [5,6,7]. Molecular analysis of its structure and binding affinities tends to confirm a specific activity on COVID-19 proteins, compared with other steroids [8]. In systemic autoimmune diseases, Prednisone remains the main used steroid, but high dosages of dexamethasone have recently been used in immune thrombocytopenic purpura, allowing a shorter course of therapy, and some authors recommend its utilization as a first-line treatment [9,10]. Consequently, the increase in dexamethasone prescription may be associated with increased frequency of DDI, as described in some observational database studies. In a large cross-sectional study of 444 elderly and diabetic Portuguese patients for instance, DDI between dexamethasone and fluoroquinolones, enhancing the risk of tendinopathy and tendon rupture, was found in 12 patients, and accounted for as much as 27% of potentially serious DDIs detected in this cohort [11]. A study of potential DDI with imatinib, performed in 544 French patients with at least one prescription of imatinib, a substrate and inhibitor of CYP3A4, revealed that dexamethasone, which was prescribed in as much as 23% of patients, was the third potentially interacting agent involved (after Paracetamol and proton pump inhibitors), leading to specific recommendations to prescribers [12]. This is consistent with a Chinese study on potential DDI with every oral antineoplastic agent (13.917 patients), which found interaction between these oral chemotherapies and dexamethasone in 117 patients (39% of every registered DDI in this study) [13]. Nevertheless, the real impact of such DDI with dexamethasone remains poorly defined, as shown in a recent review of six databases of DDI between COVID-19 treatments and, respectively, cardiovascular and antidiabetic agents, where dexamethasone was described as a “moderate risk of DDI which requires caution and close monitoring“ with all 19 anti-diabetic, anti-hypertensive and cardiovascular system-acting agents screened in this study [14]. The recent increase in the use of high doses of dexamethasone during the COVID-19 pandemic have therefore highlighted the need for better knowledge of the clinical significance of DDI involving dexamethasone in the clinical setting.

## 3. Dexamethasone Pharmacokinetics, Metabolism, and Drug Interactions

The pharmacokinetics of dexamethasone in human subjects has been extensively studied and documented in the literature. As dexamethasone is almost eliminated from plasma within 24 h, single-dose studies are representative for a once-daily dosing regimen, which is the most common mode of administration in clinical practice. Overall, glucocorticoids display high oral bioavailability varying from 60% to 100% [15]. In humans, the fraction of administered dose systemically available for oral dexamethasone is 76% ± 10%. Dexamethasone is eliminated (K_el_ = 0.16) mainly by hepatic metabolism and renal excretions of the metabolites. Plasma concentrations follow a bi-exponential pattern [15]. Dexamethasone protein binding and hepatic metabolism have been well characterized in the literature. Key intrinsic (age, sex, body weight, ethnicity) and extrinsic (smoking) factors have been investigated. The pharmacokinetic parameters of dexamethasone, as established from the literature, are summarized in Table 1. The pharmacokinetic properties of a 20 mg oral dose of NEOFORDEX^®^ (the main form of oral dexamethasone available in 2023) have been studied clinically and are summarized in Table 2.

The dexamethasone hepatic metabolism is a two-step process. Firstly, oxygen or hydrogen atoms are added then, secondly, conjugation takes place (glucuronidation or sulphation). Dexamethasone is extensively metabolized to 6-hydroxy-dexamethasone (6-OH-DEX) and side-chain cleaved metabolites in the human liver both in vitro and in vivo, with CYP3A4 responsible for the formation of 6-hydroxylated products [15]. Metabolites are excreted in the urine and the bile [16]. The renal excretion of unchanged dexamethasone is ≤10%. Dexamethasone is a well-known substrate [15] and inducer of CYP3A4 and P-gp and may therefore be subject to metabolic DDI, as noted in the CHMP “Note for Guidance on the Investigation of Drug Interactions” (CPMP/EWP/560/95). There is a reduced metabolic clearance rate (98 ± 43 l·m^2^ versus 153 ± 45 l·m^2^ daily) and prolonged plasma half-life (5.9 ± 2.2 h versus 3.5 ± 1.0 h) in patients with liver disease [17].

**Table 1:** Dexamethasone’s pharmacokinetic parameters following systemic administration (from reference [14], except for the Vd and CL of oral dexamethasone, which were adapted from reference [18]).

Drug	Route	F (%)	C_max_ (μg/L/1 mg-dose)	t_max_ (h)	t_1/2_ (h)	Vd (L) *	CL (L/h) *	k_e_ (h^−1^)
Dexamethasone after dexamethasone sodium phosphate	IV	90	10.5 ± 2.8 (10.2–10.8)		4.6 ± 1.2	65.7 ± 17.3 (27.0–98)	12 ± 4 (5–21)	0.21 ± 0.03
Dexamethasone	Oral	76 ± 10 (61–86)	8.4 ± 3.6	1.5 (1.0–2.0)	4.0 ± 0.9	76.3	7.7 (5.2–9.7)	0.16

* Volume of distribution (Vd) and total clearance (CL) parameters were normalized to 70 kg body weight; IV: intravenous; F: oral availability (% of the administered dose systemically available); C_max_: peak plasma concentration; t_max_: time to reach C_max_; h: hours; t_1/2_: terminal half-life; k_e_: elimination rate constant.

**Table 2:** Pharmacokinetic properties of a single 20 mg oral dose of dexamethasone (NEOFORDEX^®^) (adapted from dexamethasone assessment report—European Medicinal Agency, 17 December 2015, EMA/CHMP/6613/2016).

Pharmacokinetic Parameter	Arithmetic Means (±Standard Deviation *)
AUC_(0–36)_ (h·µg/L)	1116.86 (±346.20)
AUC_(0–∞)_ (h·µg/L)	1140.30 (±366.43)
C_max_ (µg/L)	125.93 (±23.06)
t_max_ (h)	3.43 (1.8–8.0)
Half-life (h)	4.60 (±1.26)

* range for t_max_.

## 4. Clinical Rationale for the Study of Pharmacokinetic Drug–Drug Interactions Involving Dexamethasone

A dose–effect relationship for glucocorticoids was first described in 1967 in patients with multiple myeloma and intermittent high-dose dexamethasone was chosen for practical reasons. There is an excellent linear relationship between oral dexamethasone dose and, respectively, AUC and C_max_. However, AUC is independent of the individual oral solid dosage form and differences in bioavailability have a minor influence on AUC. No non-clinical or clinical studies have been conducted that specifically address the question whether the total exposure or the peak exposure to dexamethasone is more important for its pharmacodynamic action in these patients. There is evidence to suggest that total exposure is the relevant factor. In the first line treatment of multiple myeloma, dexamethasone is more effective when combined with other drugs than when administered alone, and that some drug combinations including dexamethasone are more effective than others. Furthermore, in the context of specific drug combinations, dexamethasone dosing may have prognostic implications. In COVID-19, a randomized controlled study comparing 6 versus 12 mg of dexamethasone failed to identify any statistically significant difference in terms of days alive without life support [19]. However, post hoc analysis highlighted interindividual variability and suggested that a dose of 12 mg could be more beneficial in some patients, yet the presence of co-medications (except for other immunosuppressive drugs) was not studied [20]. For dexamethasone, an agent that is regularly prescribed and frequently associated with other drugs, either empirical data on differing efficacy depending on the co-medications or data on its interaction with major proteins implicated in drug metabolism, and the observed trend towards personalized adaptation of the dosage to patient-specific characteristics in a precision medicine approach, emphasizes the need to explore the clinical relevance of drug–drug interactions that could led to the choice of another glucocorticoid or, alternatively, adjustment of the recommended dosage.

## 5. In Vitro Evidence of Drug–Drug Interactions Involving Dexamethasone

(A) Induction of CYPs by Dexamethasone: Role of Nuclear Receptors
  (1) Induction of Liver CYP3A4

**Figure 1:**
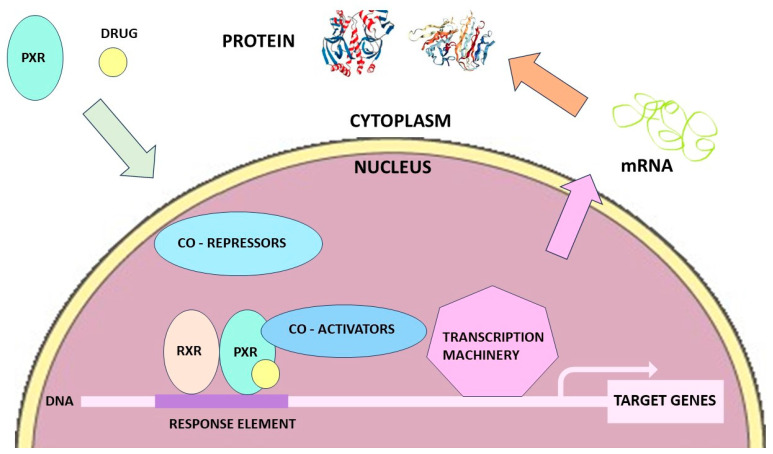
Effect of PXR on the regulation of CYP3A, CYP2C or the ABCB1 genes (the effect of hCAR on the regulation of CYP3A, CYP2B, CYP2C and transporter genes would give a similar picture), (adapted from reference [29]).

Members of the CYP3A subfamily, mainly the CYP3A4 isoform, are highly expressed in the human liver and small intestinal tract, playing a pivotal role in the systemic exposure of more than 50% of the currently marketed drugs [21]. CYP3A4 is the major drug-metabolizing enzyme in the human liver and small intestine and is responsible for the clearance of many commonly used drugs including steroids, benzodiazepines, statins, calcium channel blockers, direct oral anticoagulant medications and HIV-1 protease inhibitors. CYP3A4 has been shown to be inducible in humans by dexamethasone [22,23] Induction by a drug can affect not only the clearance of a concomitantly administered medication but also its own clearance (auto-induction) by the induced enzyme. Such induction is mediated by the binding of dexamethasone to nuclear receptors, mainly the Pregnane X receptor (PXR), the constitutive androstane receptor (CAR) and/or the Glucocorticoid receptor (GR) in a concentration-dependent manner [24]. Nuclear receptors are a class of proteins directly binding and interacting with DNA, regulating the expression of adjacent genes and acting as transcription factors. PXR and CAR are known to have an important place in the activation of several transporters and CYP family proteins. A schematic synthesis of their action is described in Figure 1. Studies conducted in several hepatocyte cell lines showed that dexamethasone was a strong PXR substrate and liver CYP3A inducer even when tested at low, nanomolar (0.01 µM), clinically relevant concentrations [25]. This study also demonstrated that dexamethasone activated the PXR promoter, which may synergistically induce CYP3A4 with profound toxicological consequences in clinical practice [25]. In summary, by using several distinct in vitro cultured hepatocytes, dexamethasone significantly enhanced CYP3A4 mRNA synthesis, protein expression and activity, starting at nanomolar concentrations and displaying a dose–response curve with fold-induction increasing with dose to a point of saturation (E_max_ usually up to a 15-fold induction) at up to 250 µM, leading to an EC_50_ of 50 to 75 µM of dexamethasone [26,27,28]. These in vitro cell culture systems appeared to be good clinical predictors of CYP3A4 induction in clinical practice; it is interesting to note that the most potent clinical inducer, i.e., rifampicin, produced the highest CYP3A4 mRNA fold-induction in immortalized hepatocytes [27]. A dual effect on the intensity of induction has been detected with two various mechanisms depending on the dexamethasone concentration. At concentrations >10 µm, dexamethasone has a direct binding effect on PXR, inducing a PXR activation which in turn induced CYP3A with a high amplitude (a factor of 15–30), whereas at sub-micromolar concentrations (i.e., more physiological conditions), the mechanism of CYP3A4 induction was a GR-dependent transactivation of PXR and/or CAR, xenobiotic-independent and of lower amplitude (a factor of 3–4) [28].

  (2) Induction of Other CYPs

Dexamethasone also induced CYP2C9 expression and activity in human hepatocytes [25,26]. The CYP2C subfamily is an important class of drug-metabolizing enzymes responsible for the metabolism of up to 20% of all currently prescribed drugs [30]. In humans, this subfamily is composed of CYP2C8, CYP2C9, CYP2C18 and CYP2C19, with CYP2C8 and CYP2C9 being expressed at the highest level in the human liver [23]. In a study performed using primary human hepatocytes, 10 µM dexamethasone enhanced CYP2C8 mRNA two-fold, through GR activation, suggesting that dexamethasone might also induce CYP2C8 expression and activity in extra-hepatic tissues [30]. These results were consistent with studies performed in hepatocyte cultures and showing that dexamethasone increased human constitutive androstane receptor (CAR) by a GR-dependent mechanism and induction of CYP3A4, CYP2C8 but also CYP2B6 [31]. An enhancement of the effects of induction by known inducers such as phenobarbital and rifampicin has also been shown, with maximum induction of CYP2C8 and CYP2C9 at 0.1 µM dexamethasone, a dose at which neither CYP3A4 nor CYP2B6 were induced, suggesting complex mechanisms of regulation, involving all three nuclear receptors (PXR, CAR and GR) simultaneously [32]. CYP2A6 is expressed predominantly in the liver, representing between 1 and 10% of total hepatic P450s and responsible of the metabolism of substrates ranging from pharmaceuticals to toxins including procarcinogens, especially nicotine-to-cotinine C oxidation in smokers. In four different primary human hepatocyte cultures, dexamethasone induced CYP2A6 mRNA up to 10-fold in a concentration-dependent manner, starting as low as 0.1 µM, through activation of the GR and interaction with hepatic nuclear factor 4α. Hence, dexamethasone-mediated induction of CYP2A6 expression and activity may have profound implications on the rate of nicotine metabolism and clearance, and hence tobacco dependence in the clinical setting [33].

(B) Regulation of Expression and Activity of Transporters by Dexamethasone: Potential Implications for Drug–Drug Interactions

**Figure 2:**
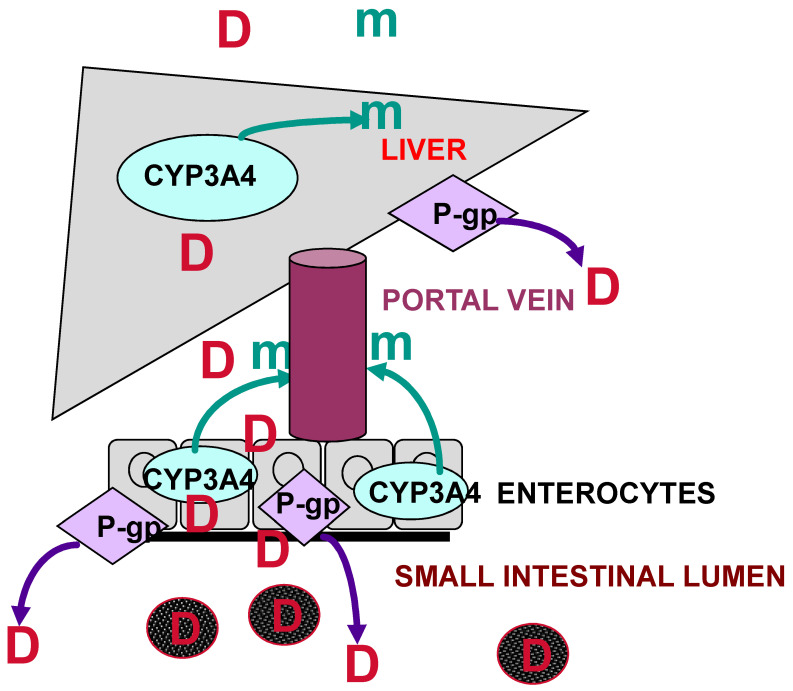
Schematic representation of combined human liver and intestinal first-pass extraction of xenobiotics (CYP3A4: cytochrome P450 3A4; P-gp: P-glycoprotein; D: drug; m: metabolite) (from reference [35]).

The modulation of expression and activity of drug-metabolizing enzymes and drug transporters by inducers is a major concern in the development of new drugs because it potentially leads to changes in the bioavailability of drugs and may disturb the balance between efficacy and toxicity [34]. Besides the liver, the small intestine expresses a broad spectrum of phase I and phase II drug metabolizing enzymes and transporters commonly referred to as phase III enzymes in drug metabolism. Phase I, II and III enzymes were shown to play a cooperative role in detoxifying and excreting xenobiotics, by liver and intestinal first-pass extraction. A comprehensive image of first-pass extraction is presented in Figure 2. Similarly to the human liver, small intestinal CYPs and transporters have been reported to be sensitive to induction [34].

  (1) The Major Role Played by P-glycoprotein

P-glycoprotein (ABCB1, P-gp) is the product of the MDR1 gene in humans and was first characterized as the ATP-dependent transporter responsible for efflux of chemotherapeutic agents from resistant cancer cells. P-gp is located within the brush border on the apical (luminal) surface of mature enterocytes and on the apical surface of hepatocytes [36]. There is wide overlapping substrate specificity between CYP3A4 and P-gp, including drugs with a narrow therapeutic index, such as terfenadine, simvastatin, lovastatin, felodipine, amiodarone and midazolam [37] Although it has not been demonstrated so far, dexamethasone may induce their metabolism and transport, thus accelerating their elimination or preventing intestinal absorption, which in turn may decrease their efficacy. Typically, if a drug undergoes significant (greater than 60%) CYP3A4 metabolism in the gut and the liver in addition to P-gp-mediated efflux, the likelihood of significant DDI increases, especially in patients taking multiple CYP3A4 and/or P-gp substrates. The well-conserved family of transmembrane proteins also includes multidrug resistance-related proteins (MRP) 1 to 6 but the clinical relevance of the latter transport proteins in DDI in humans has yet to be determined. Interactions that occur at the P-gp level may explain many non-metabolic DDIs, emphasizing the need to assess the respective role of metabolizing enzymes and transporters in drug biotransformation.

  (2) Induction of P-glycoprotein by Dexamethasone

By using human jejunum originated from surgical resections and carefully prepared in a laboratory, Van de Kerkhof EG et al. showed that 100 µM of dexamethasone incubated for 24 h led to a four-fold induction of jejunal CYP3A4 mRNA, associated with a 50% increase in CYP3A4 activity, and a two-fold induction of jejunal MDR1 mRNA, the gene encoding for human P-gp [34]. Using sandwich-cultured rat hepatocytes and Rhodamine 123 as a model P-gp substrate, some authors observed that 10–50 µM of dexamethasone added to the hepatocytes for 48 h led to a significant three-fold increase in P-gp-mediated efflux transport of the model substrate and biliary clearance, but had a lesser effect on the biliary excretion index, suggesting increased hepatocyte uptake of Rhodamine 123 through induction of another transporter in rats [38]. As observed with CYP3A4, upregulation of P-gp expression and activity by dexamethasone was dose- and time-dependent, with maximal induction obtained with 10 µM concentrations, and strongly correlated with dose-dependent PXR upregulation [39]. Collectively, the current results may have significant clinical implications. Indeed, dexamethasone-mediated P-gp induction can lead to altered pharmacokinetics and variable drug disposition of concomitantly administered medications. Of note, the regulation of P-gp by dexamethasone appeared to be tissue- or cell-specific, with some authors reporting similar increases in P-gp expression and activity in brain, retinal barrier, small intestine, lung and canalicular biliary tract, while others reported no change or decreased expression in the colon and kidney [38,39,40] or even a decrease in the kidney [40]. Additionally, a study observed a GR-dependent eight-fold P-gp mRNA induction in human lymphocytes treated with 1 µM dexamethasone, suggesting that dexamethasone may increase P-gp-mediated efflux of drugs such as antiretroviral agents, that need to enter the lymphocyte to be effective [41]. The same team observed a four-fold P-gp gene expression induction in ex vivo human cytotrophoblasts by dexamethasone and betamethasone, but not by prednisone. This result suggests that dexamethasone may decrease the maternal–fetal permeability to associated P-gp substrate medications, which may be protective or deleterious for the fetus [42]. This supports the recommendation by the French reference center to preferentially use prednisone, prednisolone or methylprednisolone over dexamethasone during pregnancy, for other reasons than obstetrical indications. In summary, the clinical implication of P-gp induction by dexamethasone in DDI in the clinical setting is not straightforward and deserves specific evaluation of each potential interaction in clinical studies.

  (3) Effect of Dexamethasone on Other Transporters and Metabolism Enzymes

Besides P-gp (the MDR1 gene product), other ABC transporters also catalyze the detoxification of xenobiotics and excretion of their conjugated metabolites in humans. In an in vitro study conducted on A549 cells (the non-small-cell lung cancer cell line) treated with dexamethasone, Pulaski L et al. showed a two-fold induction of the multidrug resistant protein MRP3 mRNA, while MRP2 expression was not induced [43]. In human embryonic kidney line-cells, dexamethasone increased MRP2- and MRP4-mediated transport of 3H-Methotrexate up to 170% and 140%, respectively [44]. These results highlight the fact that the scope of clinically significant DDI involving dexamethasone may go beyond CYP3A4 and P-gp. Breast cancer-related protein (BCRP), another ABC efflux transporter located all along the human small intestinal and canalicular biliary tracts, is responsible for the efflux of several widely prescribed medications such as methotrexate, mitoxantrone or non-steroidal anti-inflammatory drugs [45]. In a breast cancer cell line overexpressing BCRP, dexamethasone significantly decreased BCRP activity, thus increasing the entrance of mitoxantrone into the cell and its cytotoxicity [46]. Two further studies confirmed the impact of dexamethasone on the modulation of the placental barrier in pregnant women and revealed that it not only involved P-gp but also other transporters, such as modulation of BCRP transcription and activity and the increase of MRP4 expression, which may affect drug toxicity and efficacy during pregnancy [47,48].

Organic anion transporting polypeptides (OATPs in humans, Oatps in rodents) belong to a growing superfamily of ATP-independent transport proteins that mediate uptake of structurally diverse amphiphilic organic solutes [49]. To the best of our knowledge, 11 human OATPs have been identified to date and some of them (namely OATP1B1 and OATP1B3) mediate the transport of a variety of drugs including rifampicin and dexamethasone. An induction of OATP1A4 by dexamethasone, via the difference between the level of biliary excretion index and the level of P-gp induction, has been hypothesized [38] but it has not been reported elsewhere. Conversely, dexamethasone was shown to inhibit OATP1A2-mediated dehydroepiandrosterone transport [50].

In terms of metabolism, after a CYP3A-mediated biotransformation, Phase II reactions (also known as ‘conjugation reactions’) generally serve as a detoxifying step in drug metabolism by adding a hydrophilic compound to the drug. Phase II drug metabolizing enzymes are mainly transferases (UDP-glucuronosyltransferases (UGT family)), sulfotransferases (SULT family), N-acetyltransferases, glutathione S-transferases (GST family) and methyltransferases (mainly thiopurine S-methyl transferase and catechol O-methyl transferase). Induction of several of these enzymes by dexamethasone was previously described in rat hepatocytes but not in human cell lines, emphasizing the limits of in vitro cell culture systems to predict in vivo DDI involving dexamethasone and phase II metabolizing enzymes [51,52,53,54].

(C) In Vitro Interaction between Dexamethasone and Other Treatments

In vitro experiments using mice and rat primary hepatocyte cultures and human liver microsomes showed consistently that docetaxel concentrations were significantly decreased by dexamethasone, which is associated with a 50% induction of P-gp efflux and/or CYP3A-mediated docetaxel metabolism [55,56,57]. In a work on a triple negative breast cancer cell line, the combination of docetaxel and dexamethasone did not decrease the cytotoxicity of docetaxel, which was even higher than with docetaxel alone, suggesting that, despite the DDI between either drug, efficacy may not be altered in the clinical setting [57]. Similarly, when dexamethasone and doxorubicin, both drugs being substrates of P-gp and CYP3A, were simultaneously administered, a significantly higher cell survival rate was observed as compared to the cell survival rate observed with doxorubicin alone [57]. Similar unfavorable pharmacodynamic interaction was observed between dexamethasone and camptothecin (a topoisomerase inhibitor, whose derived products, irinotecan and topotecan, are widely prescribed and substrates of P-gp and CYP3A) with an increase in the cell survival rate under their combination compared to camptothecin alone [58]. Finally, no significant difference in terms of antiproliferative activity was detected with adjunction of dexamethasone to cisplatin and docetaxel on a carcinoma of head and neck cell lines [59]. In the FDA-approved anti-vascular endothelial growth factor tyrosine kinase tivozanib intrinsic clearance was increased by 60% with concomitant administration of dexamethasone [60]. However, the clinical implication of such pharmacokinetically significant DDI has yet to be determined. In terms of rheumatic diseases, a recent study on the dual-drug interaction between dexamethasone and the immunosuppressive Janus kinase inhibitor tofacitinib, suggests a possible gender-dependence of interaction, finding a synergistic effect on the lymphocyte proliferation inhibition in male lymphocytes, but an antagonistic effect in female lymphocytes, both in humans and rats [61].

(D) In Vitro Interaction with Dexamethasone as a CYP3A4 and P-gp Substrate

Besides its induction property, dexamethasone is also a substrate of CYP3A and P-gp. Therefore, DDI affecting the efficacy and toxicity of dexamethasone may occur. The level of its efflux by P-gp, estimated using transfected cell lines, showed an efflux-ratio of 3.7 compared to 1 without the presence of P-gp, consistent with the definition of a moderate substrate as compared to other corticosteroids [62]. However, cyclosporine A, at concentrations used in rheumatoid arthritis patients in clinical practice, may dose-dependently enhance the intracellular uptake of dexamethasone by inhibition of P-gp-mediated efflux, as shown in a human leukemic resistant cell line [63]. In another study using human liver microsomes to explore three statins (atorvastatin, rosuvastatin and Fluvastatin) and their ability to activate PXR, CYP2A6, 2B6 and 3A4, dexamethasone-induced GR activity, assessed by luciferase-activity, was dose-dependently antagonized by several fluvastatin enantiomers, suggesting a potential risk of efficiency decreasing by co-administration between fluvastatin and dexamethasone [64]. In human cancer cell lines and human hepatocytes, proton pump inhibitor lansoprazole, but not omeprazole, activated PXR and GR and induced GR-dependent luciferase activity by dexamethasone from 27- to 97-fold [65].

(E) In Vitro Studies General Conclusion

Collectively, these in vitro studies highlighted the role of dexamethasone in the regulation and modulation of the expression and activity of numerous transporters in human cancer cell lines and normal tissues. The activity of dexamethasone, as a substrate, may vary. Whether or not these findings may be extrapolated in vivo in humans is currently unknown and may be hard to conclusively determine given the species differences observed by some authors between rats and humans [66]. However, future studies will be warranted to confirm the major and growing role of uptake transporters in the drug–drug interactions that may occur with high-dose dexamethasone.

## 6. In Vivo Evidence of Drug–Drug Interactions Involving Dexamethasone

(A) Relative Contribution of CYP3A4 and P-gp in Drug–Drug Interactions Involving Dexamethasone in Animal Studies

The consequences of exposure to dexamethasone on the expression and function of liver and small intestinal CYP3A4 and on the expression and efflux of P-gp were previously evaluated in rats [67]. Compared to controls, CYP3A4 expression and activity (as measured by triazolam hydroxylation) in rat liver microsomes was increased by 10 to 14-fold by dexamethasone, with high correlation between CYP3A4 protein expression and activity. Intestinal P-gp protein expression was increased by 2.8-fold, while P-gp expressed in brain microvessels only increased 1.3-fold following dexamethasone treatment [67]. Changes observed in the expression and activity of liver and intestinal CYP3A4 and P-gp, at both mRNA and protein levels, were probably responsible for the cyclosporine-dexamethasone drug–drug interaction observed in rats treated with 1 or 75 mg/kg of dexamethasone once a day for 1–7 days. In this study, total cyclosporine clearance was unchanged but oral bioavailability was decreased by 30% after 7 days of treatment with 1 mg/kg dexamethasone. In rats treated with 75 mg/kg dexamethasone, the concentration of cyclosporine in the blood was significantly decreased after intravenous and oral administration, respectively. Cyclosporine bioavailability also decreased and total clearance significantly increased, consistent with a drug–drug interaction occurring at the liver and small intestine levels [68]. The respective role of P-gp and CYP3A in these mechanisms of absorption was studied in the intestine, with mdr1−/− knockout mice, without expression of P-gp. Expression of either gene’s mRNA was not homogenously distributed in the rat intestine, CYP 3a being more expressed in the upper intestine, than in the lower; MDR1 expression, being the exact opposite and that in physiological conditions; CYP3A expression in the upper intestine is the main determinant of cyclosporine absorption [69]. It has been shown that, after treatment with 1 or 75 mg/kg of dexamethasone, the oral bioavailability of cyclosporine was 43 and 25% in wild-type mice, respectively, but increase to 89 and 73% of the control in knockout mice. Whereas the induction of CYP3A was observed only with high-dosage dexamethasone in the intestine. This suggests that, unlike in physiological conditions as stated above, P-gp become a major determinant of cyclosporine absorption in the intestine after induction by dexamethasone [70]. Based on these results, we assume that patients treated with cyclosporine, and are candidates to receive high doses of dexamethasone, should be carefully monitored regarding their cyclosporine trough concentrations. Pre-treatment of rats with 40 mg/kg/day of dexamethasone for three days had no effect on the systemic exposure of intravenous indinavir, a well-known CYP3A4 and P-gp substrate [71]. However, when 20 mg/kg of indinavir was given orally to pre-treated rats, dexamethasone decreased C_max_ 10-fold, increased t_max_ two-fold, decreased indinavir systemic exposure (as measured by the AUC) three-fold and decreased oral bioavailability from 28% to 12%, which is consistent with a strong involvement of indinavir pre-systemic small intestinal first-pass induction in this drug–drug interaction with dexamethasone [71]. The effects of dexamethasone in decreasing the AUC of several molecules have been reported in more recent studies with several new drugs, known CYP3A substrates in mice and/or rats: a 39% decrease in AUC of tyrosine kinase inhibitor erlotinib [72]; an 85% and 91% decrease in AUC of triptolide and (5R)-5-hydroxytriptolide, an active agent of *Tripterygium wilfordii*, a Chinese plant used in rheumatisms [73]; a 47% decrease in the AUC of loxoprofen, a cyclooxygenase inhibitor, with the formation of an (OH)-loxoprofen metabolite [74]; a 90% decrease in AUC of abiraterone acetate, a prostate cancer hormonotherapy [75]. Interestingly, the same extent of AUC decrease (56%) was reported with widely used antalgic nefopam in a rat population, the metabolism of which is not fully elucidated; however, suggesting the involvement of CYP3A [76].

The role of P-gp has also been emphasized in a study on nadolol, a non-metabolized beta blocker, conducted in rats and showing that dexamethasone, given orally at 8 mg/kg/day for 4 days increased the P-gp levels two-fold in the liver and small intestine, which is consistent with previous findings, and decreased its systemic exposure by one-third, and increased its P gp-mediated renal excretion almost two-fold, which is consistent with a major role of P-gp in the DDI [77]. In rats pretreated with 100 mg/kg/day of dexamethasone given orally for two days, P-gp expression increased two-fold in the intestine, as previously observed, but not in the liver. In vitro metabolism studies in microsomal suspensions indicated a 9.7-fold increase in liver CYP3A activity, consistent with dexamethasone induction. In vivo, dexamethasone increased Rhodamine 123 intestinal efflux two-fold, in good agreement with Western Blot analysis. In pre-treated rats, the inhibitory potencies of midazolam, a CYP3A substrate and modulator, and verapamil, a CYP3A and P-gp substrate and modulator, decreased both in the liver and small intestine due to dexamethasone induction of P-gp and CYP3A in rats [78].

A mechanism-based PK/PD model was developed to characterize the complex concentration-induction response relationship between dexamethasone and CYP3A and to resolve the drug- and system-specific PK/PD parameters for the course of induction [79]. A two-compartment model with zero-order absorption was applied to describe the pharmacokinetic characteristics of dexamethasone. The maximum induction of CYP3A mRNA via PXR transactivation by dexamethasone was achieved, showing a 21.3-fold increase relative to the basal level. The CYP3A protein was increased eight-fold and the total enzyme activity was increased almost three-fold, as previously described, with a lag-time of 40 h from the t_max_ of the dexamethasone plasma concentration [79]. These results were consistent with another work in which an oral and intravenous in vivo CYP3A probe (^13^C-erythromycin breath test) [80] was used with or without dexamethasone, in order to describe dexamethasone-mediated CYP3A induction in rats by means of a physiologically based pharmacokinetic model [81]. The clinical significance of such interaction is not always obvious but in a veterinary study dexamethasone interfered with the pharmacokinetics of ivermectin, a well-known P-gp substrate widely used in humans and veterinary medicine. This DDI, studied in young cattle, and presumably involving ivermectin P-gp-mediated intestinal transport, was associated with the decreased clinical efficacy of ivermectin (based a less-effective reduction of fecal egg count) and lower plasma concentrations (as characterized by a 40% reduction of AUC) [82].

Another important property of P-gp is its crucial role at the blood–brain barrier. [83] The brain benzodiazepine receptor binding of oral midazolam was significantly reduced by pre-treatment with 80 mg/kg of dexamethasone 24 h before oral midazolam administration, due to liver and small intestinal CYP3A induction in rats, which was consistent with the 80% decreased plasma concentrations of midazolam. These results were consistent with previous findings showing that dexamethasone shortened the sleeping time induced by midazolam, in addition to significant decreases in the midazolam plasma concentrations in rats. Hence, this study demonstrated for the first time, by means of the brain benzodiazepine receptor occupancy measurement, the potential pharmacodynamic consequences of this pharmacokinetic DDI between dexamethasone and oral midazolam [84]. Dexamethasone also decreased misonidazole neurotoxicity in mice by significantly increasing drug clearance and shortening drug half-life, presumably through the induction of misonidazole liver metabolism and/or elimination, although the latter assumption was not verified in the study. Interestingly, the diminution of AUC in the brain (a 57% reduction) was superior to its diminution in blood (a 16% reduction), as compared to the control [85]. To our knowledge, no study on P-gp efflux or expression was performed with any molecule, despite the fact that several studies confirmed an induction of P-gp at the blood-brain barrier level [86]. Consistent with in vitro findings, P-gp and CYP3A induction by dexamethasone are the two mainly described determinants of DDI in vivo in animal studies, although the possibility of other independent mechanisms with some other targets cannot be ruled out.

(B) Drug-Drug Interactions with Other Transporters in Animals

One study conducted in rats showed that dexamethasone reduced methotrexate biliary excretion by 53% and potentiated its hepatotoxicity in rats without affecting methotrexate pharmacokinetics and systemic exposure, presumably through the induction of uptake transporters located at the basolateral membrane of the hepatocytes (Oatp1a4 and Oat2), although upregulation of the efflux ABC transporter MRP2 and downregulation of MRP3, located in the canalicular (apical) membrane of the hepatocytes, were also observed in this study [87]. Whether these results should be extrapolated to humans deserves further confirmation. Inhibition of BCRP by dexamethasone in the placenta was also confirmed in a study on pregnant mouse placenta. A dose-dependent inhibition of BCRP at a high-dose of dexamethasone (1 mg/kg) with decreased mRNA levels and protein function, as attested by the accumulation of substrates in the fetus [88].

(C) Interaction by Unknown or Unspecified Mechanisms

In a mouse model of cysticidal infection, albendazole treatment efficacy (a CYP3A4 but not P-gp substrate) was significantly reduced by co-administration of dexamethasone at an inflammatory disease equivalence dosage, as attested by the number of alive parasites [89]. The exact mechanism of such DDI is unknown, as albendazole pharmacokinetics parameters are increased in the presence of concomitant dexamethasone treatment [90]. In an in vivo model of human ovarian carcinoma xenocraft on nude mice, a premedication with dexamethasone reduced the inhibitory effect of paclitaxel, another taxane family antitumor agent, on tumor growth by approximately 20% [91]. In a model of rheumatoid arthritis, a synergistic effect was observed between tofacitinib and dexamethasone, on paw growth with a 0.76 interaction factor [92]. Taken together, these studies indicate that dexamethasone-mediated induction of CYP3A-mediated metabolism and transport of widely used medications in various therapeutic areas may significantly influence clinical outcomes.

## 7. Drug–Drug Interactions Involving Dexamethasone in Humans

(A) Clinical Rationale

As mentioned above, members of the CYP3A subfamily of drug metabolizing enzymes are the most abundantly expressed cytochrome P450 enzymes in the human liver and small intestinal tract and are involved in the metabolism of more than 50% of clinically used medications. In addition, because many drugs, including some used in chemotherapy regimens, are metabolized in whole or in part by the CYP3A system, the determination of the role of dexamethasone in DDI observed in humans and the extent of CYP3A induction by dexamethasone in vitro and in vivo is important, because the relative role of well-described CYP3A inducers, such as rifampicin or carbamazepine, is well-described in the literature and they are known perpetrators of DDIs. Descriptions of clinically relevant DDIs involving dexamethasone is sparse and, as underlined by some authors, the majority of potential DDIs reported in databases are only theoretical [93]. In adults, hepatic CYP3A activity reflects primarily the net contributions of CYP3A4 and CYP3A5, which share overlapping substrate specificities but differ in terms of expression and transcriptional regulation [94]. Recent efforts to understand inter-individual variability in CYP3A activity have focused primarily CYP3A5 genetic polymorphisms since variability in the contribution of functional CYP3A5 activity could influence an individual’s susceptibility to inducer- or inhibitor-mediated DDI. The major CYP3A5 polymorphisms include the CYP3A5*3, *6 and *7 alleles which are functionally inactive, while the CYP3A5*1 allele is the only functional allele known to contribute to total CYP3A activity [94]. The CYP3A5*1 allele is known to differ among ethnic groups and has been associated with higher midazolam systemic clearance and tacrolimus dose requirements in adults, and a interindividual difference in terms of susceptibility to DDI with dexamethasone might be supposed [94].

(B) The Role of CYP3A Activity and P-gp in Drug–Drug Interactions Involving Dexamethasone in Humans: Published Evidence

As the influence of the CYP3A5 genetic polymorphisms on the extent of DDI involving CYP3A inducers is unknown, a clinical study was conducted in 27 healthy volunteers, half of whom were carrying the functional CYP3A5*1 allele and the half were not. The CYP3A activity was measured by means of the ^14^C-Erythromycin Breath Test, a specific and validated probe for liver CYP3A activity [80]. In this study, dexamethasone, 8 mg given orally twice daily, significantly increased the CYP3A activity by 50% in non-carriers of the CYP3A5*1 allele, while CYP3A was not significantly induced in those carrying the functional allele [95]. Hence, the risk of DDI involving dexamethasone in clinical practice may depend on the CYP3A5 genetic polymorphism that is not routinely determined except in some organ transplant recipients treated with tacrolimus, a well-known substrate of liver and small intestinal CYP3A4/5. Lapatinib, an orally administered chemotherapeutic agent for the treatment of metastatic breast cancer is predominantly metabolized by CYP3A4. In nested case-control studies involving 120 patients with metastatic breast cancer treated with lapatinib, patients receiving the combination were 4.57 times (95% CI, 1.23–16.88, *p* = 0.02) more likely to develop hepatotoxicity and 3.48 times (95%CI, 1.24–9.80, *p* = 0.02) more likely to develop a clinically important change in liver enzymes, as compared to patients who did not receive the combination [96]. These results, obtained from a retrospective study, were ascribed by an in vitro study showing a 59% decrease in hepatocytes’ viability in a cell culture system treated with the combination. Collectively, these findings provided substantial evidence and insights into the clinical relevance of dexamethasone-lapatinib DDI, through CYP3A induction, in increasing lapatinib-induced hepatotoxicity in humans. Panobinostat, a histone deacetylase inhibitor used in the treatment of refractory multiple myeloma, is being widely prescribed in combination with dexamethasone. Approximately 40% of Panobinostat undergoes liver CYP3A4-mediated metabolism [97]. In a phase Ib study evaluating the combination of panobinostat with bortezomib and dexamethasone (20 mg given orally) in the treatment of multiple myeloma, the authors observed a 20% reduction of panobinostat systemic exposure as compared to a cycle without dexamethasone [98]. However, the overall response rate was superior in patients receiving the combination with dexamethasone.

Dexamethasone induction of CYP3A4, but also P-gp activity, may be dose-dependent, as low doses of dexamethasone (1.5 mg/day for 4 days) did not induce triazolam metabolism and systemic exposure in ten healthy volunteers [99], while higher doses of dexamethasone (10 mg/day for 2 days) decreased cyclophosphamide systemic exposure by almost 50%, in nine patients treated with high-dose cyclophosphamide before bone marrow transplantation [100]. Likewise, dexamethasone given orally to 18 healthy volunteers at a dose of 4 mg for only one day did not interact with the cyclosporine analog and the well-known P-gp substrate valspodar [101]. In addition, valspodar did not significantly affect dexamethasone’s pharmacokinetics in this study. The new direct oral anticoagulants (apixaban, rivaroxaban, edoxaban and dabigatran) are substrates of P-gp, and all but dabigatran undergo CYP3A4-mediated liver and small intestinal metabolism. The possible occurrence of clinically relevant DDI in the context of COVID-19 has been a major concern, as most hospitalized patients received high doses dexamethasone, concomitant with oral anticoagulants in some patients. In a recent study, no change in the peak and trough plasma concentrations of 26 patients treated with anticoagulants (apixaban, rivaroxaban and edoxaban) was observed when measured 48 to 72 h after the initiation of dexamethasone and 14 to 21 days after cessation [102] A nested case–control study from the National COVID Cohort Collaborative on 172 patients who received dexamethasone and rivaroxaban or apixaban and 344 controls without dexamethasone, found no increase in thromboembolic events under treatment with dexamethasone [103].

(C) Drug–Drug Interactions Involving Dexamethasone with Other CYPs in Humans

Dexamethasone may also interact with some other CYP substrates, such as CYP2C9, CYP2C8 or CYP2B6, which were shown to be inducible by dexamethasone. Voriconazole, a major antifungal agent approved in the treatment of invasive aspergillosis, is a substrate of multiple CYP450 isoenzymes mainly including CYP2C19, CYP2C9 and CYP3A4. Some case reports have reported treatment failure with voriconazole administered to cure invasive fungal infection, associated with suboptimal concentration of voriconazole due to coadministration with dexamethasone, consistent with a previous pharmacokinetic study showing that dexamethasone decreased voriconazole trough concentrations at suboptimal therapeutic levels [104,105,106]. Interestingly, a recent pharmacogenetic study revealed that the CYP2C19*17 “gain-of-function” allelic variant [107], associated with increased enzymatic activity, was associated with an even more significant decrease in C_min_ induced by glucocorticoid [106].

Among the various side effects associated with the use of the antiseizure drug phenytoin, severe thrombopenia is rather unusual and mostly occurred in patients concomitantly treated with dexamethasone [108,109,110,111,112,113]. Phenytoin is metabolized through aromatic hydroxylation catalyzed by the CYP2C9 (90%) and CYP2C19 (10%) isoforms. The reactive intermediate, epoxide may be responsible for such severe side effect and, even if conflicting data exist, most published evidence showed that dexamethasone administration resulted in decreased phenytoin concentration, consistent with induced CYP2C9/19-mediated metabolism [108,109,110,111,112,113].

(D) Dexamethasone Pharmacokinetics May Also Be Affected by Well-Known and Potent CYP3A/P-gp Modulators in Humans

The pharmacokinetics of dexamethasone may also be affected by CYP3A4 and/or P-gp modulators, some of which are widely prescribed by physicians in various therapeutic areas in clinical practice. A randomized crossover study versus placebo conducted in healthy volunteers receiving 4.5 mg oral or 5 mg intravenous dexamethasone revealed that the potent CYP3A4/P-gp inhibitor itraconazole markedly increased dexamethasone plasma concentrations and enhanced its adrenal-suppressant effect when given at 200 mg/day [114]. In this study, the systemic exposure of oral and intravenous dexamethasone was increased 3.5-fold, together with a three-fold increase in the elimination half-life. Intestinal absorption of oral dexamethasone was also 50% faster with itraconazole, as ascribed by the 50% decreased T_max_, consistent with possible inhibition of intestinal P-gp-mediated efflux of the steroid [114]. In addition, the morning plasma cortisol concentration was suppressed after dexamethasone administration for at least two days longer in the presence of usual therapeutic doses of itraconazole, consistent with a clinically relevant DDI. Oral bioavailability of dexamethasone was, however, only slightly increased from 75% to 86%, suggesting systemic rather than pre-systemic enzyme inhibition to explain this drug interaction [114].

In clinical practice, the antiemetic drugs from the neurokinin-1 (NK1) receptor antagonist family, i.e., Aprepitant and fosaprepitant, are weak CYP3A4/P-gp inhibitors, and often concomitantly prescribed with dexamethasone in various chemotherapy associations. Aprepitant, given at 125 mg on day 1, then 80 mg on days 2 to 5 increased the systemic exposure of dexamethasone by two-fold at day 1 and day 5. The authors therefore recommended dexamethasone dose adjustment in patients treated with aprepitant [115]. Fosaprepitant, given intravenously at a dose of 150 mg/day, increased the systemic exposure of dexamethasone, given orally (8 mg/day) for 3 days, two-fold [116]. As with aprepitant, the authors suggested that the dexamethasone dosing regimen should be decreased by 50% in the presence of fosaprepitant, as ascribed in a recent retrospective study on patients with lymphoma treated with a chemotherapy combination including dexamethasone (R-DHAP) [117]. Interestingly, dexamethasone clearance was only decreased by 30% when concomitantly administered intravenously with oral aprepitant, suggesting limited interaction only at the level of the liver but not intestinal CYP3A4 [118].

To our knowledge, no study with other well-known liver and intestinal CYP3A/P-gp inducers (e.g., phenobarbital, primidone, carbamazepine, rifampicin, aminogluthetimide and rifabutin) or inhibitors (ketoconazole, itraconazole, posaconazole, voriconazole, ritonavir, nelfinavir, erythromycin, clarithromycin and telithromycin) has ever shown that such decreased efficacy or increased toxicity in humans, respectively, at dosages used in hematology or cancerology population. Some clinical case reports have, however, been described with low-dose dexamethasone. Primidone, an anti-seizure drug known as a potent inducer of CYP3A4, CYP2C9 and CYP2C19, and also P-gp, have demonstrated clinically significant DDIs with dexamethasone in two children with congenital adrenal hyperplasia and taking either medication concomitantly. In one patient, introduction of primidone led to an eight-fold increase in dexamethasone dosing regimen to achieve clinical efficacy [119]. In the second case, primidone withdrawal led to dexamethasone overdosage, further requiring a three-fold reduction in the dexamethasone dosing regimen [120]. Finally, interference with the 1 mg dexamethasone suppression test was described with co-administration of the well-known potent CYP3A4/P-gp inducers, rifampicin, carbamazepine and troglitazone in previously published reports [121,122,123]. Such clinically relevant interactions, including the clinical consequences and the currently available level of evidence are summarized in Table 3.

(E) Impact of Pharmacogenomics on the Potentiality and Prediction of Dexamethasone Induced DDIs

As previously published [95,106], genetic background can significantly influence the occurrence and clinical relevance of DDI. A growing number of single nucleotide polymorphisms (SNPs) have been identified and are currently studied to predict inter-individual variability of drug efficacy and toxicity. However, despite evidence of the role of genetic background in the occurrence of DDI’s, even suggesting drug-drug-gene interactions [124], only a few pharmacogenomic-based studies have proved to be useful in individualizing drug treatment and dosing regimen, e.g., anti-platelet agents or anti-depressant drugs [125,126], or reducing the incidence of clinically relevant adverse drug reactions [127]. A modern approach with bioinformatics tools, combining a larger panel of genes and a faster computerized approach, may help the clinician to reduce the risk of DDI, although generalization and the feasibility of such an approach has yet to be determined [128,129]. Regarding dexamethasone, the genes encoding for transport proteins and metabolizing enzymes involved in its liver first-pass and pre-systemic metabolism are commonly studied. CYP3A5*1, for instance, is an SNP present in up to 80% of humans of African American descent and confers functional CYP3A5 activity in this population, in addition to CYP3A4 [94]. Tacrolimus dose adjustment is based on CYP3A5-based genetic polymorphism in transplant recipients in clinical practice [130]. Whether patients with a functional CYP3A5*1 allele may be less sensitive to dexamethasone induction is currently unknown [95]. These patients may indeed be less sensitive to dexamethasone induction of tacrolimus metabolism, thus requiring lower or no dose adjustment as compared to patients who do not carry the allele. However, in some cases the genotyping of CYP3A5 failed to clearly reflect the activity of CYP3A, being, for instance, unable to predict lapatinib-induced hepatotoxicity [131]. Despite a relatively minor allele frequency (MAF) of 3–5%, the main genetic variant of CYP3A4, i.e., CYP3A4*22, which directly affects CYP3A4 activity, failed to predict interindividual variability of various substrate concentrations [132,133].

Other clinically relevant SNPs have been described for the metabolizing enzymes CYP2C8, CYP2C9, CYP2B6 and CYP2C19. However, to the best of our knowledge, no association with the extent of dexamethasone metabolism or inductor potency have been specifically detected, except for the aforementioned work in which CYP2C19*17 SNP enhanced the decrease in Voriconazole by dexamethasone induction of enzymatic activity [106]. CYP2C19*17 is the main “gain-of-function” SNP for this CYP. Single nucleotide polymorphisms were also described in the transporters’ genes, especially in the ABCB1 gene encoding for P-gp [134]. Single nucleotide polymorphisms in the genes of nuclear receptors [135] were also described. The role of these various SNPs in predicting the dexamethasone level of enzyme or transporter induction in humans is still unknown and will be presumably challenging in the future of personalized medicine. Indeed, to date, the best-described and relevant SNPs do not concern the proteins involved in dexamethasone pharmacokinetics and metabolism/transport, but rather pharmacodynamics and targets such as chaperone or the glucocorticoid receptor [136,137,138,139,140]. However, specific association with ABCB1 genetic polymorphisms (rs1128503, rs2032582 and rs1045642) and dexamethasone, but not prednisone or hydrocortisone efficacy, has been observed in children with congenital adrenal hyperplasia [137]. A trend toward increased time to progression for carriers of the ABCB1 SNP (rs2229109), as compared to non-carriers, was observed in patients with multiple myeloma treated with dexamethasone and lenalidomide [141]. As no impact of ABCB1 on lenalidomide systemic exposure and efficacy was observed in a separate study [142], the results presented in the Falk et al. study [141] may be attributed to dexamethasone systemic exposure variation. Finally, association between pharmacokinetic parameters and the efficacy of dexamethasone have only been studied in patients with multiple myeloma and those with leukemia [143,144].

## 8. Overall Conclusions

Dexamethasone has well-established clinical indications, involving both systemic and local administration, regarding its anti-inflammatory, immunosuppressant and antineoplastic properties. There is strong in vitro and in vivo evidence that dexamethasone, at a clinically relevant dosing regimen, e.g., doses used in the treatment of COVID-19 infection or hematologic malignancies, is a potent inducer of CYP3A4, CYP2C9, P-gp, and presumably other transporters, as confirmed in several studies including human studies or case reports. Despite the lack of warnings regarding the clinical relevance of such DDIs in daily practice, prescribers should be aware that dexamethasone, especially at the high dosages used clinically, may significantly alter the efficacy and safety of drugs with a narrow therapeutic index, such as substrates of CYP3A4 and P-gp, as has recently been observed in COVID-19 patients treated with the antiviral nirmatrelvir and concomitantly receiving potent CYP3A4/P-gp inducers [145]. Moreover, as dexamethasone is a substrate of CYP3A4, it may also be susceptible to DDI when administered with strong inducers or inhibitors such as rifampicin, phenytoin or carbamazepine [146,147]. Better knowledge of an individual patient’s pharmacogenetic background and a pharmacogenomic-based approach may be useful in anticipating DDI between individual drugs in the clinical setting. The recent substantial increase in the use of dexamethasone in clinical practice during the COVID-19 pandemic has highlighted the urgent need of clinical DDI studies to optimize dosing regimens in patients with comorbidities and concomitant medications.

## Figures and Tables

**Table 3:** Examples of clinically relevant and severe drug–drug interactions with dexamethasone in humans with clinical implications.

Drugs Implicated	Time to Occurrence	Dexamethasone Dosage *	Nature of Interaction	Clinical Implication	Quality of Evidence
Lapatinib	Days to weeks	Low to high	Induction of lapatinib CYP3A-mediated metabolism: exposition to reactive, potentially toxic metabolites of lapatinib	Hepatotoxicity leading to lapatinib withdrawal in some cases	Moderate
Panobinostat	Few days	High	Decrease in panobinostat plasma concentrations by up to 20%	Possibly better disease control under a combination of dexamethasone and Panobinostat	Moderate
Cyclophosphamide	Few days	High	Decrease in Cyclophosphamide plasma concentrations	Theoretical decreased cyclophosphamide efficacy although no available signal so far	Low
Voriconazole	Few days	High to moderate	Decreased voriconazole plasma concentrations due to CYPC19, CYP2C9 and CYP3A induction	High to moderate increase in the risk of treatment failure, thus requiring voriconazole therapeutic drug monitoring	Moderate
Itraconazole	Few days	Moderate	Increase in dexamethasone plasma concentration by itraconazole mediated CYP3A4 inhibition	Potential increase in adrenal suppression at day 4, scant data currently available regarding potentially prolonged adrenal suppression	Moderate
Aprepitant/fosaprepitant	Immediate	High	Increase in dexamethasone plasma concentration by itraconazole mediated CYP3A4 inhibition	Low clinical implication due to sequential treatment and anticipated lowering of dexamethasone posology in the guidelines	Moderate
Phenytoine	few days	High	Induction of phenytoine metabolism by CYP2C9/CYP2C19	High, thrombopenia with fatal cases reported	Very low
Primidone	<1 month	Low	Decrease in dexamethasone activity by metabolism induction	Moderate, lack of control of congenital adrenal hyperplasia	Very low
rifampicin	Immediate	Low	Decrease in dexamethasone activity by metabolism induction	Moderate, misdiagnosis of Cushing syndrome	Very low
carbamazepine					Very low
troglitazone					Very low

*: Low doses did not exceed 1 mg, intermediate dosage ranged from 5 to 10 mg, as observed in patients hospitalized with COVID-19 infection, and high dosages were 20 mg and above, usually observed in cancer patients.

## Data Availability

Data are contained within this article.
